# Fabrication of Elastic Color-Changing Films—Elastomer Films Incorporating Mechanochromic Fluorenylidene–Acridane

**DOI:** 10.3390/molecules30081761

**Published:** 2025-04-14

**Authors:** Koki Iwasaki, Yutaka Matsuo

**Affiliations:** 1 Department of Chemical Systems Engineering, Graduate School of Engineering, Nagoya University, Nagoya 464-8603, Aichi, Japan; 2 Institute of Materials Innovation, Institutes for Future Society, Nagoya University, Nagoya 464-8603, Aichi, Japan

**Keywords:** mechanochromism, fluorenylidene–acridane, elastomer, polyacrylate, swelling, mechanical pressure

## Abstract

A pressure-sensitive elastomer film incorporating fluorenylidene–acridane (FA) in its folded conformation was successfully developed for use in pressure-sensitive applications. The elastomer network was swollen with acetone, creating space to accommodate FA molecules. Although FA dissolved in acetone and adopted a twisted conformer, a solvent exchange process with methanol facilitated the reprecipitation of FA in its yellow folded conformation within the elastomer matrix. Confocal and scanning electron microscopy confirmed the incorporation of FA in its folded form within the matrix, while film stretching testing and water resistance analyses highlighted the film’s durability. The film exhibited a reversible color change upon mechanical pressure, reverting back to yellow when treated with methanol. This approach presents a promising method for the integration of FA into elastomer films, with potential applications in flexible mechanical sensors and other responsive materials.

## 1. Introduction

**Figure 1:**
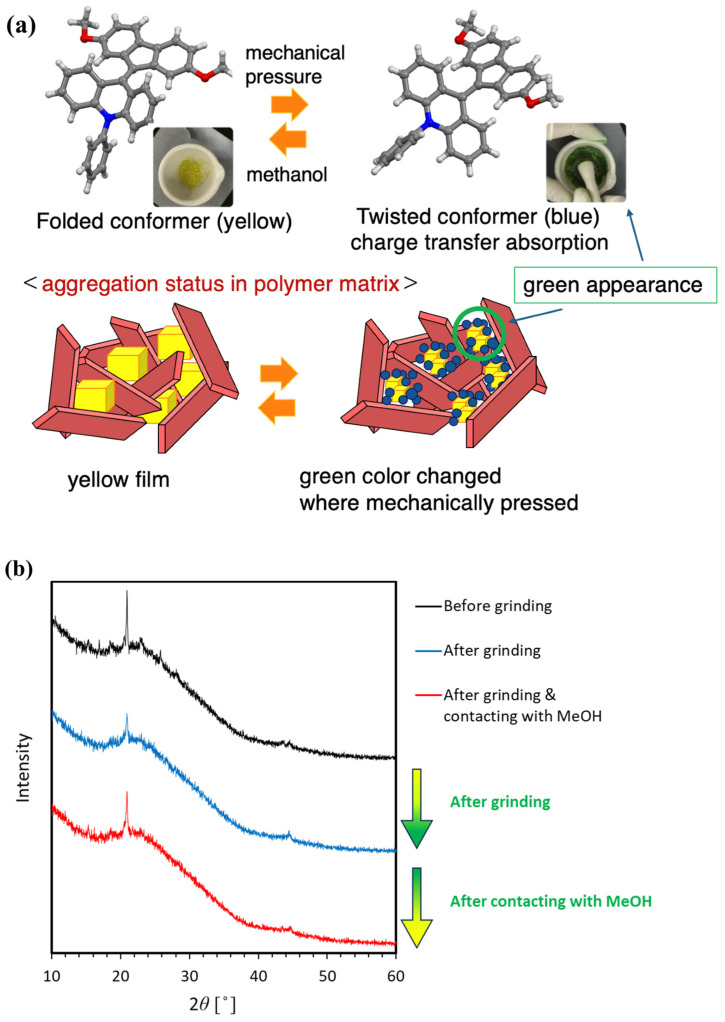
Fluorenylidene–acridane (FA) with mechanochromic conformational change used in this work. (**a**) Basic properties. (**b**) Powder XRD spectra of the state before and after grinding of pristine folded FA.

Mechanochromic materials exhibit changes in their properties upon the application of mechanical stimuli, making them highly promising for a variety of applications, including pressure sensing, recording, and display devices [1,2,3,4,5,6,7,8,9,10,11,12,13,14,15,16,17,18]. Mechanochromism can be divided into two categories: excited-state mechanochromism, which involves changes in emission color change, and ground-state mechanochromism, which involves changes in absorption color change [19,20,21,22,23,24,25,26,27]. In this study, we utilized fluorenylidene–acridane (FA, Figure 1a), a rare ground-state mechanochromic material discovered in our laboratory [1,2,3,4,5,6]. This material, which showed mechanochromism based on conformational change, was rare among mechanochromic materials because usually mechanochromism is caused by a difference in morphology. This material demonstrates a remarkable absorption color change from yellow to dark green upon exposure to mechanical pressure. The dark green color is due to a mixture of the yellow folded conformer and the blue twisted conformer. This color change is attributed to alterations in the molecular aggregation and conformational change. When the molecule was dissociated with mechanical stimuli, the molecular interaction and the conformation changed based on energetic stability. Then, charge transfer absorption was caused from the electron-donating acridone part to the electron-withdrawing fluorene part, leading to these visual color change [1,2,3,4,5,6]. When mechanical pressure disrupts molecular aggregation and some molecules become isolated, conformational change from the folded conformer to the twisted conformer occurs, causing the color change from yellow to green. This conformational change was also caused when dissolved in good-solubility solution based on absorption spectra of FA in various solvents [3]. Furthermore, the conformational changes and aggregation revert upon contact with alcohol, resulting in the original yellow color [1,2,3,4,5,6]. The XRD spectra of FA imply that folded FA showed crystallinity, and twisted FA did not show it (SmartLab, Rigaku Holdings Corp., Akishima, Japan). This was because pristine FA showed a sharp peak, and it attenuated after grinding, except for one peak, which became sharp again just after coming into contact with methanol (Figure 1b). Another quantitative evaluation of FA, such as the identification of single crystal structures and crystal morphology calculation, was reported in previous research through X-ray analysis of its single crystal [3].

To harness these properties, we developed pressure-sensitive films using FA [6]. The pressure-sensitive films enabled measurement of the mechanical pressure and visualization of the pressure distribution based on the intensity of the color change [6]. So far, we have reported a paper soaked with FA [2], vacuum-evaporated film [4], cellulose nanofiber (CNF) film [6], and polyvinyl alcohol film [4] containing FA inside. Mechanical pressure on the films was transferred to the FA embedded inside the films, and the mechanochromism of the FA led to a visual film color change with this transferred pressure. Herein, we report an elastic film containing mechanochromic FA. After several attempts to include the yellow folded conformer of FA into the elastic film, we successfully fabricated mechanochromic elastic films that change color to green upon mechanical stimuli. The resulting film exhibited high stretchability, a mechanical pressure response, reversible color changes, and excellent water resistance. This study highlights the potential for further advancements in mechanochromic film-based devices utilizing FA, paving the way for innovative applications in pressure sensing and beyond [28,29,30].

## 2. Results and Discussion

**Figure 2:**
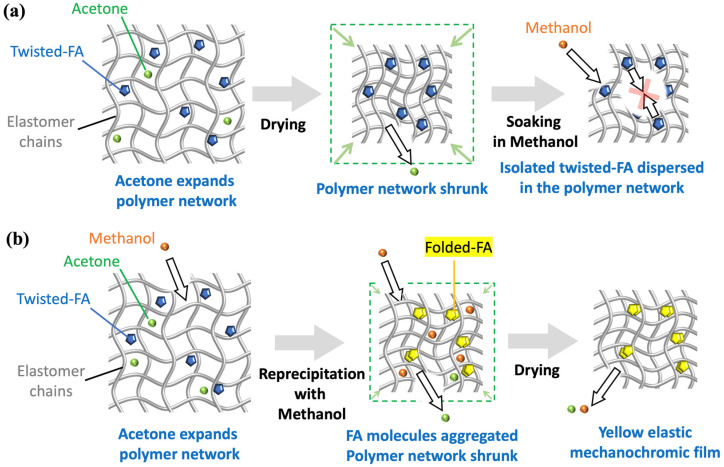
The concept of a swelling elastomer network to make space for the accommodation of FA molecules. (**a**) Polymer shrunk before aggregation of FA molecules to afford blue films. (**b**) Successful process to obtain yellow elastic mechanochromic films with aggregation of FA molecules through reprecipitation with methanol within swollen polymer network.

The elastomer used in this study was an acrylic polymer in which 95% of the carboxyl groups in its side chains were esterified with alkyl groups and further treated with a crosslinking agent. In this study, we investigated a method to incorporate FA into the elastic film based on the expansion and contraction mechanism of elastomer polymer chains (Figure 2) [31,32,33,34]. The critical point here was to incorporate FA into the elastomer film while maintaining its yellow folded conformer. This is essential because the yellow film serves as the starting point for the color change from yellow to green.

First, several solvents, including xylene, chloroform, acetone, and isopropanol, with the Hansen solubility parameter (HSP) ranging from 8.0 to 12.0, were selected to compare the swelling degree of the elastomer sheet in each solvent (see Section 3) [35,36]. Among these, acetone exhibited the highest swelling ratio, achieving a 41% increase in size, indicating that it was the optimal solvent for swelling the elastomer sheet. Subsequently, we confirmed that the expansion ratio of the sheet swollen in acetone was nearly identical to its shrinkage ratio after drying. Moreover, both the swollen and shrunken sheets retained their original shape, with the edge length remaining consistent, suggesting the isotropic expansion of the sheet in acetone.

**Figure 3:**
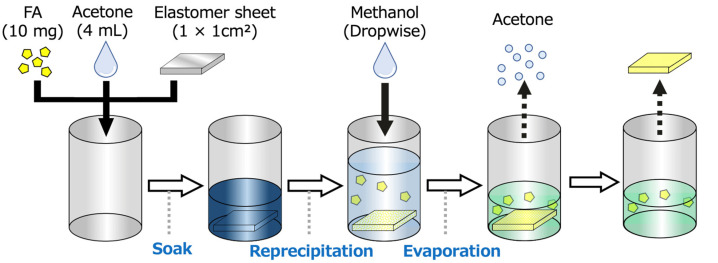
Fabrication of FA-containing elastomer films with swelling in acetone and reprecipitation with methanol.

Next, we investigated the inclusion of FA in its folded form within the elastomer film. Acetone effectively expands the space within the polymer network, enabling it to accommodate FA molecules. However, FA dissolves in acetone, and in this solvent, isolated FA molecules adopt a twisted conformer. When both the yellow powder of FA and the elastomer film were soaked in acetone, the solution turned blue, indicating that the FA molecules had changed to their twisted conformer. The elastomer film also appeared blue, suggesting that the twisted FA conformer was incorporated into the film. The blue film was then taken out from the solution and dried to shrink the film. However, this process yielded a shrunken blue film, indicating that the FA conformer did not revert to its folded form. To address this, we attempted to reprecipitate FA within the elastomer network by using solvent exchange (Figure 3). Methanol was added to the blue solution containing the elastomer film, and acetone was removed using a rotary evaporator. This solvent exchange, replacing the good solvent (acetone, bp = 56 °C) with a poor solvent (methanol, bp = 65 °C), caused the solution to change color from blue to green and resulted in the formation of a yellow elastomer film. During this process, the dissolved twisted FA reprecipitated within the polymer network of the elastomer into microcrystalline FA in its folded form. This configuration is essential for mechanical pressure-sensing experiments. As a result, the mass of this fabricated film and of the original film without FA were, respectively, 6.1 mg, and 5.5 mg. Accordingly, given that the manufactured film did not include any solvent particle, it was described as 9.84 wt% elastomer–FA film.

**Figure 4:**
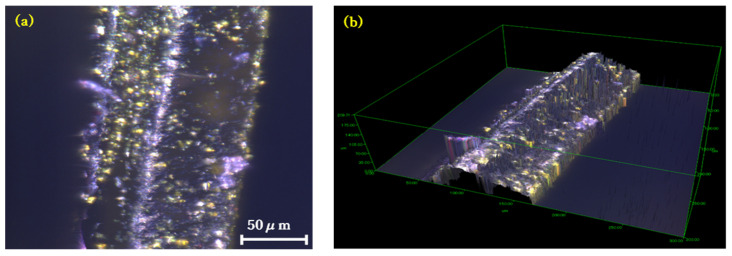
Confocal microscopy images. (**a**) Cross-sectional view. (**b**) Three-dimensional view. Yellow granules indicate FA in its folded conformation.

**Figure 5:**
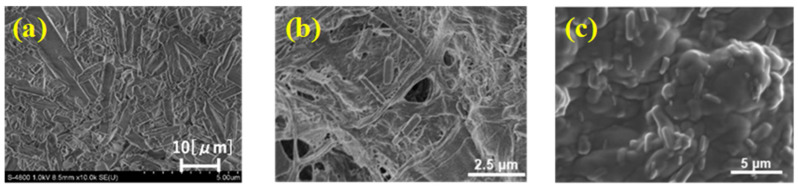
SEM images of film surfaces. (**a**) FA/elastomer film. (**b**) FA/CNF film [larger-fiber-diameter CNF]. (**c**) FA/CNF film [smaller-fiber-diameter CNF].

The fabricated elastomer film was evaluated for its pressure responsivity, elasticity, cross-sectional and surface morphology, and water resistance. The cross-section and surface of the fabricated film were observed using confocal microscopy (OPTELICS, Lasertec Corp., Yokohama, Japan, Figure 4) and scanning electron microscopy (SEM, S-4800, Hitachi High-Tech. Corp., Tokyo, Japan, Figure 5). Cross-sectional images confirmed the presence of FA in its folded conformation within the film. Additionally, SEM images revealed that the porosity of the FA/elastomer film was lower than that of our previous FA-containing CNF film. This explains the necessity of swelling the film with acetone by 41% enlargement to accommodate FA into the polymer network. The surface smoothness analysis showed a slightly rougher FA/elastomer surface compared with the original elastomer film (see Section 3). The prolonged immersion of the sheet in liquid may have compromised its surface smoothness. Overall, surface roughness levels of the present FA/elastomer and our previous FA/CNF films were at the same level.

The water resistance of each film was evaluated by measuring the time-dependent change in the water contact angle (see Section 3) [37]. The elastomer film exhibited a high initial contact angle, which remained consistently higher than the values observed for FA/CNF films over time. This result demonstrated the excellent water resistance and durability of the elastomer film. The hydrophobic nature of the elastomer, derived from its chemical structure, and the low porosity observed in the SEM images were likely contributors to this water resistance.

**Figure 6:**
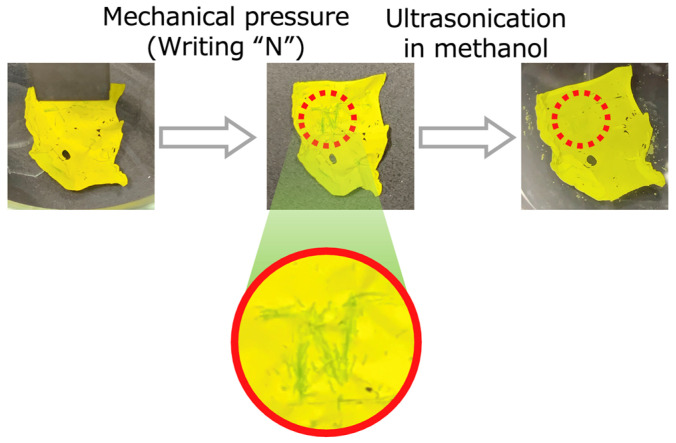
Mechanical pressure response of FA-containing elastomer film. Color changing by mechanical stimuli from yellow to green and reversible color change back to yellow by ultrasonication in methanol.

Mechanical pressure responsiveness was examined by scratching the film with sharp tweezers and observing the color change upon contact with methanol. Partial color changes from yellow to green and reversibility were confirmed in the scratched areas (Figure 6). Note that mechanochromism was not observed with heat (60 °C), and that heating for a long time led to melting the base material, namely the elastomer. Furthermore, the intensity of the color change in the scratched region was quantitatively evaluated through image analysis. The cyan content, which is indicative of the proportion of twisted FA conformers, was hypothesized to correlate with the intensity of the observed color. Given that twisted FA exhibits a blue hue, its proportion likely influences the cyan content. Using a colormath module in the Python program, the cyan content in the scratched regions was calculated to evaluate the relationship between color intensity and applied mechanical pressure [6]. The results showed a cyan content of 0.0% in the unscratched area and 10.5% in the scratched area. Thus, the cyan content of the FA/elastomer film is expected to vary between approximately 0–10% under different mechanical pressure levels. Furthermore, the higher concentration of FA embedded inside the film, the stronger the mechanical pressure responsiveness expected. This is because more folded FA particles would be distributed near the film surface. The absorption color of the FA/elastomer film changed with the characteristics of the FA. The visual light reflectance of this film was expected to decrease as the mechanical pressure increased because the scratched spot had a darker color than an unscratched area. In reference to the UV-Vis spectra obtained from the FA films in the previous research, the spectrum peak would shift to a shorter wavelength in the range of 500–600 nm as the pressure increased. In the previous research, the peak reflectance of the FA/CNF films decreased by approximately 20% on average under 300 MPa pressure. However, the FA/elastomer film had a weaker pressure response, so it was speculated that the amount of decrease would be less than 20% in the same situation [6]. Next, the color reversibility of the film was observed. A bit of methanol was added and ultrasonicated on the scratched film. Then, the added methanol was removed with a measuring pipette after ultrasonication. As a result, a color change back to yellow was confirmed with ultrasonication of this film in methanol.

**Figure 7:**
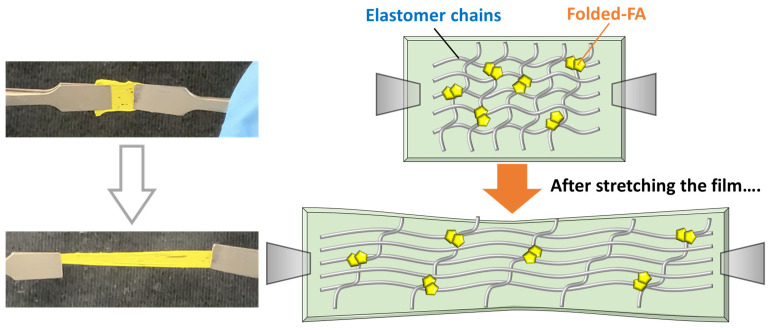
Examination on elasticity of FA/elastomer sheet, and speculation on network condition in each state.

Finally, when the fabricated film was stretched uniaxially using two tweezers, it demonstrated sufficient elasticity. This result was attributed to the minimal stress transmitted to the FA particles due to the elastomer’s inherent flexibility. Furthermore, no color change was observed whilst stretching the film, which meant that the embedded FA did not show mechanochromism during film stretching. From a structural perspective, it was expected that each FA particle was fixed in this polymer network, and that the aggregated FA particles were not dissociated but moved as they were (Figure 7). However, it was also anticipated that when this elastomer was stretched and the tensile strain surpassed a specific value, a color change would happen and the film color intensity would increase. This speculation was based on previous studies on polymer gel, which changed color with changes in its network structure [38,39].

## 3. Materials and Methods

### 3.1. Materials

Dimethoxy fluorenylidene–acridane, 9-(9*H*-fluoren-9-ylidene)-10-(4-methoxyphenyl)-9,10-dihydroacridine, denoted as FA in this paper, was synthesized according to our previous paper [2]. Elastomer films were supplied from Osaka Organic Chemical Industry Ltd. (Osaka, Japan). This elastomer is a standard acrylate polymer containing alkyl esters on 95% of the carboxylic acid groups, and the polymer is cross-linked. Other chemicals were purchased from commercial suppliers (Sigma-Aldrich (St. Louis, MO, USA), TCI (Tokyo, Japan), Wako (Tokyo, Japan)) and used without further purification.

### 3.2. Elastomer Films Used in This Work

In this study, the following elastomer sheets were used to evaluate their physical properties. The fundamental mechanical properties of each elastomer—including tensile strength, elongation at break, and elastic modulus—were measured using a tensile testing machine (TENSILON RTG-1310, A&D Co., Ltd., Tokyo, Japan), with film thickness = 50 μm, Young modules = 0.60 MPa, stretch rate = 1400%, and hysteresis loss rate (consumed energy/deformation energy) = 0.40%. These data provide a basis for understanding the intrinsic characteristics of the elastomers and are essential for subsequent analyses involving solvent interactions and swelling behavior.

In addition to mechanical testing, the swelling degree of each elastomer was also investigated (Table 1). This parameter was evaluated by measuring the volumetric change in the elastomer samples after immersion in a specific solvent for a defined period. The swelling degree serves as an important indicator of solvent resistance and chemical stability, and it is particularly relevant for applications where solvent permeability or material durability are a concern.

The swelling degree was calculated using Equation (1), where Q (%) represents the swelling degree. In this equation, *ρ*_2_ (g cm^−3^) and *ρ*_3_ (g cm^−3^) denote the densities of the solvent and the elastomers, respectively, and $W_{a}$ (mg) and $W_{b}$ (mg) are the weight of the elastomer sheet before and after swelling, respectively.

(1) $$Q=\frac{\rho_{3}\left(W_{b}-W_{a}\right)}{\rho_{2}W_{a}}\times 100$$

### 3.3. Fabrication of FA-Containing Elastomer Films

The specific fabrication process for the FA/elastomer film is as follows. First, elastomer sheets were cut into 1 × 1 cm^2^ squares and immersed in an FA/acetone solution (10.0 mg FA in 4 mL acetone) in a 200 mL two-neck flask. The flask was maintained at 56 °C for 48 h to enhance FA solubility and facilitate its infiltration into the elastomer sheets. Next, 200 mL of methanol was gradually added at a rate of 0.5 drops per second, and acetone was selectively evaporated using a rotary evaporator to promote the reprecipitation of FA. The solution color transitioned from blue to green, and the sheets floated at the liquid–air interface. The sheets were then retrieved, washed twice with methanol, placed on polystyrene Petri dishes, and air-dried at room temperature for one day. Through this process, the FA/elastomer films were successfully prepared.

### 3.4. Surface Smoothness Analysis

**Figure 8:**
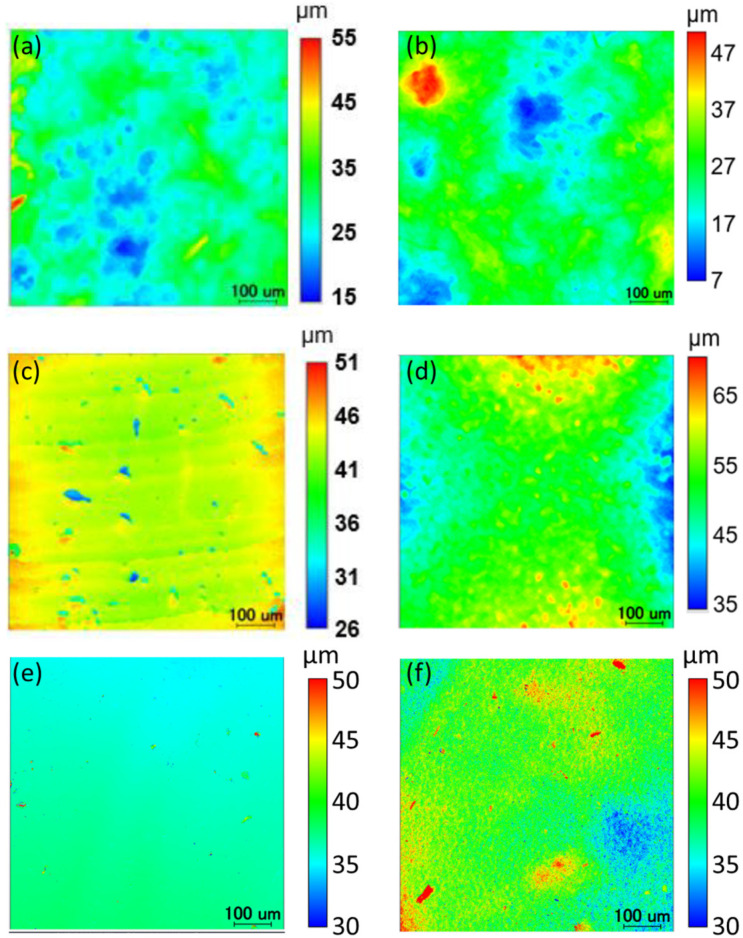
Roughness distributions of each film with a color map. (**a**) Elastomer film. (**b**) FA/Elastomer film. (**c**) CNF film (large fiber diameter). (**d**) FA/CNF film (large fiber diameter). (**e**) CNF film (small fiber diameter). (**f**) FA/CNF film (small fiber diameter).

Surface characterization was also conducted for the prepared samples (Table 2, Figure 8). The three-dimensional surface morphology of the films was analyzed by optical measurement using a confocal microscope (OPTELICS, Lasertec Corporation). Height distribution maps of each film surface were obtained as color maps. In addition, surface roughness parameters, including arithmetic mean height (*R*_a_), and root mean square height (*R*_q_), were measured as indicators of surface roughness. *R*_a_ represents the average of the absolute values of surface height deviations from the reference plane in the *z*-axis direction, while *R*_q_ is the square root of the mean of the squared height deviations from the reference. These values were measured at three different regions, with three cross-sectional measurements taken within each region. The reported values are the averages of the nine measurements.

### 3.5. Wettability Analysis

**Figure 9:**
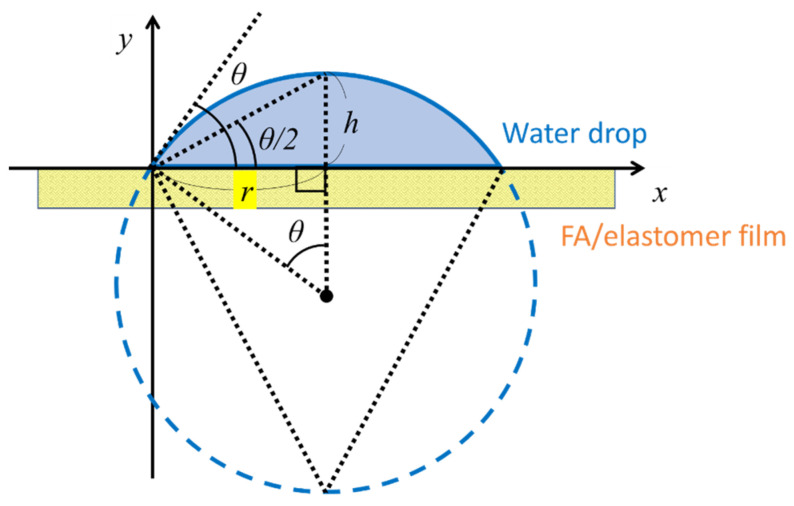
Water contact angle measurements.

**Figure 10:**
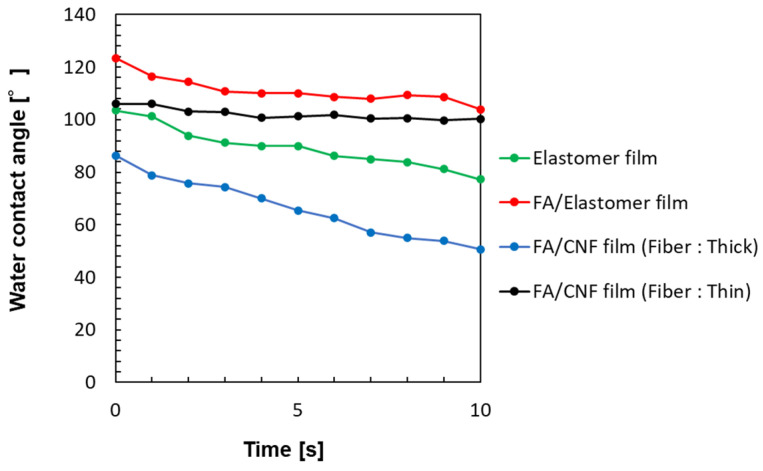
Graphic presentation of the time-dependent changes in water contact angle after dropping 2 μm water.

Water resistance was evaluated by examining the change in the water contact angle over time (Figure 9 and Figure 10, Table 3). In line with previous research, a 2 μL drop of pure water was placed on the film sample, and the change in the water contact angle was observed and recorded for 10 s. The height *h* and radius *r* of the water drop were measured, and the water contact angle was derived using the measured values and the Equation (2).

(2) $$\theta =2\;{Tan}^{-1}\left(\frac{h}{r}\right)$$

### 3.6. Pressure Responsiveness

Images of the scratched film were divided into fine pixels, and rectangular regions containing 240,000 pixels were extracted from both scratched and unscratched areas. The cyan content of each pixel was then calculated, and the average values for the regions were determined.

## 4. Conclusions

In conclusion, we successfully developed an elastomer film incorporating FA in its folded conformation, an essential development for pressure-sensitive applications. The film was fabricated by swelling the elastomer network with acetone, which created the necessary space to accommodate FA molecules. We ensured that FA remained in its folded conformation, as this yellow form is critical for triggering a color change from yellow to green upon pressure application. Acetone initially dissolved FA, causing it to adopt a twisted conformer, which was then reprecipitated into the elastomer matrix by solvent exchange with methanol. This process resulted in a yellow elastomer film containing FA in its folded conformation, suitable for mechanical pressure sensing.

The fabricated film exhibited excellent properties, including pressure responsiveness, elasticity, water resistance, and favorable surface morphology. Confocal and scanning electron microscopy confirmed the incorporation of FA in its folded form within the elastomer matrix, while surface smoothness and water resistance analyses demonstrated the film’s durability. Furthermore, the film displayed a reversible color change from yellow to green when subjected to mechanical pressure, with the color reverting back to yellow upon ultrasonication treatment in methanol. This work presents a promising approach to incorporating FA into elastomer films for use in pressure-sensitive devices, with potential applications in flexible sensors and other responsive materials.

## Figures and Tables

**Table 1:** Mass change in elastomer sheets in each solvent, and the difference in swelling degree.

	Xylene	Chloroform	Acetone	Isopropanol
HSP [-]	8.80	9.21	9.77	11.13
Density *ρ*_2_ [g/mL] (Solvent)	0.860	1.471	0.789	0.784
$\mathrm{Mass}\;\mathrm{before}\;\mathrm{swelled}\;W_{a}$ [mg] (Elastomer film)	5.3	6.3	5.2	5.5
$\mathrm{Mass}\;\mathrm{after}\;\mathrm{swelled}\;W_{b}$ [mg] (Elastomer film)	5.4	6.7	6.6	5.6
Swelling degree *Q* [%]	2.63	5.18	40.94	2.78

**Table 2:** Measured roughness parameters.

Sample	Arithmetic Mean Roughness, *R*_a_ [μm]	Root Mean Square Roughness, *R*_q_ [μm]
Elastomer	1.23	1.54
FA/Elastomer	1.61	1.99
CNF (large fiber)	3.92	4.81
FA/CNF (large fiber)	2.11	2.56
CNF (small fiber)	0.80	1.02
FA/CNF (small fiber)	0.72	0.93

**Table 3:** Time-dependent changes in water contact angle after dropping 2 μm water (unit: deg.).

Film Sample	Time [s]										
	0	1	2	3	4	5	6	7	8	9	10
Elastomer film only	103.5	101.3	93.9	91.3	90.0	90.0	86.2	85.0	83.8	81.2	77.3
FA/Elastomer film	123.4	116.4	114.3	110.7	110.1	110.1	108.6	107.9	109.4	108.7	103.9
FA/CNF film (Fiber: thick)	86.3	78.8	75.8	74.4	70.0	65.5	62.5	57.0	55.0	53.9	50.6
FA/CNF film (Fiber: thin)	106.0	106.0	103.1	102.9	100.7	101.3	101.8	100.4	100.6	99.7	100.3

## Data Availability

All relevant data are within the manuscript. The data that support the findings of this study are available in the ESI^†^ of this manuscript.

## References

[1] Suzuki T., Okada H., Nakagawa T., Komatsu K., Fujimoto C., Kagi H., Matsuo Y. (2018). A Fluorenylidene-Acridane That Becomes Dark in Color upon Grinding-Ground State Mechanochromism by Conformational Change. Chem. Sci..

[2] Matsuo Y., Wang Y., Ueno H., Nakagawa T., Okada H. (2019). Mechanochromism, Twisted/Folded Structure Determination, and Derivatization of (*N*-Phenylfluorenylidene)acridane. Angew. Chem. Int. Ed..

[3] Wang Y., Ma Y., Ogumi K., Wang B., Nakagawa T., Fu Y., Matsuo Y. (2020). Equilibrium and Thermodynamic Studies of Chromic Overcrowded Fluorenylidene-Acridanes with Modified Fluorene Moieties. Commun. Chem..

[4] Ogumi K., Nagata K., Takimoto Y., Mishiba K., Matsuo Y. (2022). Quantitative and High-resolution Mechanical Pressure Sensing Functions of Mechanochromic Fluorenylidene-Acridane. J. Mater. Chem. C.

[5] Ogumi K., Nagata K., Takimoto Y., Mishiba K., Matsuo Y. (2022). Inkjet Printing of Mechanochromic Fluorenylidene-Acridane. Sci. Rep..

[6] Ogumi K., Arakawa T., Okudera B., Takimoto Y., Nagata K., Mishiba K., Abe E., Ueno T., Hieda J., Omote K. (2024). Color-Changing Paper: Cellulose Nanofiber Films Incorporating Mechanochromic Fluorenylidene-Acridane. ACS Appl. Eng. Mater..

[7] Tang C. (2024). Fundamental Aspects of Stretchable Mechanochromic Materials: Fabrication and Characterization. Materials.

[8] Gavale R., Khan F., Misra R. (2025). Polymorphism in Mechanochromic Luminogens: Recent Advances and Perspectives. J. Mater. Chem. C.

[9] Goyal H., Kumar P., Gupta R. (2023). Polycyclic Aromatic Hydrocarbon-based Soft Materials: Applications in Fluorescent Detection, Gelation, AIEE and Mechanochromism. Chem. Asian J..

[10] Shimizu M., Nishimura K., Mineyama M., Terao R., Sakurai T., Sakaguchi H. (2024). Bis(tricyclic) Aromatic Enes That Exhibit Efficient Fluorescence in the Solid State. Molecules.

[11] Yamada K., Adachi Y., Ohshita J. (2024). Controlling the Thermodynamic Stability of Conformational Isomers of Bistricyclic Aromatic Enes by Introducing Boron and Silicon Atoms. Chem. Sci..

[12] Yang S.Y., Zhang Y.L., Kong F.C., Yu Y.J., Li H.C., Zou S.N., Khan A., Jiang Z.Q., Liao L.S. (2021). π-stacked donor-acceptor molecule to realize hybridized local and charge-transfer excited state emission with multi-stimulus response. Chem. Eng. J..

[13] Clough J.M., Weder C., Schrettl S. (2021). Mechanochromism in Structurally Colored Polymeric Materials. Macromol. Rapid Commun..

[14] Oyefusi A., Chen J. (2020). Mechanical approaches to dynamic, reversible mechanochromism based on thin film interference. Appl. Mater. Today.

[15] Jeong S.Y., Hwang W.S., Cho J.Y., Jeong J.C., Ahn J.H., Kim K.B., Hong S.D., Song G.J., Jeon D.H., Sung T.H. (2019). Piezoelectric device operating as sensor and harvester to drive switching circuit in LED shoes. Energy.

[16] White M.A. (1998). The Chemistry behind Carbonless Copy Paper. J. Chem. Educ..

[17] Tanioka M., Kamino S., Muranaka A., Ooyama Y., Ota H., Shirasaki Y., Horigome J., Ueda M., Uchiyama M., Sawada D. (2015). Reversible Near-Infrared/Blue Mechanofluorochromism of Aminobenzopyranoxanthene. J. Am. Chem. Soc..

[18] Micheletti C., Soldati L., Weder C., Pucci A., Clough M.J. (2024). Mechanochromic Polyolefin Elastomers. ACS Appl. Polym. Mater..

[19] Wu Y., Cheng X., Hu H., Hu S., Ma Z.M., Ma Z.Y. (2024). Impact of Polymer Matrix on Polymer Mechanochromism from Excited State Intramolecular Proton Transfer. Chin. J. Chem..

[20] Ito H., Saito T., Oshima N., Kitamura N., Ishizaka S., Hinatsu Y., Wakeshima M., Kato M., Tsuge K., Sawamura M. (2008). Reversible Mechanochromic Luminescence of [(C_6_F_5_Au)_2_(µ-1,4-Diisocyanobenzene)]. J. Am. Chem. Soc..

[21] Balch A.L. (2009). Dynamic Crystals: Visually Detected Mechanochemical Changes in the Luminescence of Gold and Other Transition-Metal Complexes. Angew. Chem. Int. Ed..

[22] Tsuchimoto M., Hoshina G., Yoshioka N., Inoue H., Nakajima K., Kamishima M., Kojima M., Ohba S. (2000). Mechanochemical reaction of polymeric oxovanadium(IV) complexes with Schiff base ligands derived from 5-nitrosalicylaldehyde and diamines. J. Solid State Chem..

[23] Ito H., Muromoto M., Kurenuma S., Ishizaka S., Kitamura N., Sato H., Seki T. (2013). Mechanical stimulation and solid seeding trigger single-crystal-to-single-crystal molecular domino transformations. Nat. Commun..

[24] Sagara Y., Mutai T., Yoshikawa I., Araki K. (2007). Material design for piezochromic luminescence: Hydrogen-bond-directed assemblies of a pyrene derivative. J. Am. Chem. Soc..

[25] Lavrenova A., Balkenende D.W.R., Sagara Y., Schrettl S., Simon Y.C., Weder C. (2017). Mechano- and Thermoresponsive Photoluminescent Supramolecular Polymer. J. Am. Chem. Soc..

[26] Chi Z.G., Zhang X.Q., Xu B.J., Zhou X., Ma C.P., Zhang Y., Liu S.W., Xu J.R. (2012). Recent advances in organic mechanofluorochromic materials. Chem. Soc. Rev..

[27] Fan M.Y., Cheng Y., Fang B., Lai L.M., Yin M.Z. (2021). Multicolor mechanochromism of a phenothiazine derivative through molecular interaction and conformational modulations. Dyes Pigments.

[28] Wang H.P., Zhou D.B., Cao J.G. (2014). Development of a Skin-Like Tactile Sensor Array for Curved Surface. IEEE Sens. J..

[29] Yu X.W., Mahajan B.K., Shou W., Pan H. (2017). Materials, Mechanics, and Patterning Techniques for Elastomer-Based Stretchable Conductors. Micromachines.

[30] Filippova O.V., Maksimkin A.V., Dayyoub T., Larionov D.I., Telyshev D.V. (2023). Sustainable Elastomers for Actuators: “Green” Synthetic Approaches and Material Properties. Polymers.

[31] Katashima T. (2021). Rheological studies on polymer networks with static and dynamic crosslinks. Polym. J..

[32] Wang F.S., Kruse B.J., Dickenson J.C., Zhukhovitskiy A.V. (2024). Supramolecular Templation of Entanglements and Their Spectroscopic Detection in Polymer Elastomers. Macromolecules.

[33] Watanabe M., Takeda Y., Maruyama T., Ikeda J., Kawai M., Mitsumata T. (2019). Chain Structure in Cross-Linked Polyurethane Magnetic Elastomer Under a Magnetic Field. Int. J. Mol. Sci..

[34] Safeeda N.V., Gopinathan J., Indumathi B., Thomas S., Bhattacharyya A. (2016). Morphology and hydroscopic properties of acrylic/thermoplastic polyurethane core–shell electrospun micro/nano fibrous mats with tunable porosity. RSC Adv..

[35] Mazan J., Leclerc B., Galandrin N., Couarraze G. (1995). Diffusion of Free Polydimethylsiloxane Chains in Polydimethylsiloxane Elastomer Networks. Eur. Polym. J..

[36] Kamagata K. (1958). Methods for Determining the Degree of Swelling. Nippon. Gomu Kyokaishi.

[37] Gu H.Y., Wang C., Gong S.J., Mei Y., Li H., Ma W.M. (2016). Investigation on contact angle measurement methods and wettability transition of porous surfaces. Surf. Coat. Technol..

[38] Cao Y., Wang D., Zhang Y., Li G., Gao C., Li W., Chen X., Chen X., Sun P., Dong Y. (2024). Multi-Functional Integration of Phosphor, Initiator, and Crosslinker for the Photo-Polymerization of Flexible Phosphorescent Polymer Gels. Angew. Chem. Int. Ed..

[39] Zhu Y., Pan M., Ma L., Wang Y. (2025). The luminescence mechanism of two pure organic room temperature phosphorescent isomers, mechanical force detection, 3D modeling, and dynamic data encryption. Chem. Eng. J..

